# Continuing to Confront COPD International Patient Survey: Economic Impact of COPD in 12 Countries

**DOI:** 10.1371/journal.pone.0152618

**Published:** 2016-04-19

**Authors:** Jason Foo, Sarah H. Landis, Joe Maskell, Yeon-Mok Oh, Thys van der Molen, MeiLan K. Han, David M. Mannino, Masakazu Ichinose, Yogesh Punekar

**Affiliations:** 1 Health Outcomes, GlaxoSmithKline, Uxbridge, United Kingdom; 2 GlaxoSmithKline, Real World Evidence, Uxbridge, United Kingdom; 3 University of Ulsan College of Medicine, Asan Medical Centre, Seoul, South Korea; 4 University of Groningen, University Medical Centre Groningen, Groningen, The Netherlands; 5 Division of Pulmonary and Critical Care, University of Michigan, Ann Arbor, Michigan, United States of America; 6 University of Kentucky College of Public Health, Lexington, Kentucky, United States of America; 7 Tohoku University Graduate School of Medicine, Sendai, Japan; 8 GlaxoSmithKline, Value Evidence Outcomes, Uxbridge, United Kingdom; Public Health England, UNITED KINGDOM

## Abstract

**Background:**

The Continuing to Confront COPD International Patient Survey estimated the prevalence and burden of COPD across 12 countries. Using data from this survey we evaluated the economic impact of COPD.

**Methods:**

This cross-sectional, population-based survey questioned 4,343 subjects aged 40 years and older, fulfilling a case definition of COPD based on self-reported physician diagnosis or symptomatology. Direct cost measures were based on exacerbations of COPD (treated and those requiring emergency department visits and/or hospitalisation), contacts with healthcare professionals, and COPD medications. Indirect costs were calculated from work loss values using the Work Productivity and Activity Impairment scale. Combined direct and indirect costs estimated the total societal costs per patient.

**Results:**

The annual direct costs of COPD ranged from $504 (South Korea) to $9,981 (USA), with inpatient hospitalisations (5 countries) and home oxygen therapy (3 countries) being the key drivers of direct costs. The proportion of patients completely prevented from working due to their COPD ranged from 6% (Italy) to 52% (USA and UK) with 8 countries reporting this to be ≥20%. Total societal costs per patient varied widely from $1,721 (Russia) to $30,826 (USA) but a consistent pattern across countries showed greater costs among those with increased burden of COPD (symptoms, health status and more severe disease) and a greater number of comorbidities.

**Conclusions:**

The economic burden of COPD is considerable across countries, and requires targeted resources to optimise COPD management encompassing the control of symptoms, prevention of exacerbations and effective treatment of comorbidities. Strategies to allow COPD patients to remain in work are important for addressing the substantial wider societal costs.

## Introduction

Chronic obstructive pulmonary disease (COPD) is a multifactorial and heterogeneous disease [1,2], and a global health problem afflicting millions of people worldwide [3]. It is characterised by persistent and progressive airflow limitation, the severity of which is affected by exacerbations of COPD and the presence of comorbid conditions [1]. In 2010, The Global Burden of Disease Study ranked COPD as the third leading cause of mortality and the ninth leading cause of disability-adjusted life years lost [4,5].

The substantial burden of COPD is associated with a significant economic impact both in terms of direct (healthcare and medical) and indirect (impact on home and work productivity) costs [1]. Previous international surveys have reported significant economic consequences of COPD across many developed and developing countries [6–8]. In the original Confronting COPD International Survey in North America and Europe the majority of direct costs across countries were associated with hospitalizations and medication costs [6]. In that study, annual societal costs were greater in patients with more severe disease and in those who reported comorbidities. Similarly, the BREATHE study, conducted in 11 countries across the Middle East, North Africa, and Pakistan, reported increased medical resource use in association with more severe disease, exacerbations of COPD, a higher COPD assessment Test (CAT) score and the presence of comorbidities [7]. In the COPD Uncovered International Survey of a working age population with COPD, work productivity and the level of work impairment was worse with increasing age and disease severity, and respondents reporting a greater number of comorbidities were more likely to retire early [8]. Data generated from these types of studies are important for understanding the true economic burden of COPD and may have important implications for targeting resources and informing public health policies [9].

The Continuing to Confront COPD International Patient Survey was conducted globally across 12 countries as a follow-up of the original Confronting COPD International Survey [10], and estimated the prevalence and burden of COPD [11]. This paper presents the analysis of the cost associated with COPD from the Continuing to Confront COPD International Patient Survey, including the direct and indirect costs of COPD and represents one of the largest global cost of COPD evaluations to date.

## Materials and Methods

The study design and methodology of the Continuing to Confront COPD International Patient Survey have been reported previously [11]. This was a population-based, cross-sectional survey of adults 40 years and older who fulfilled a case definition of COPD by meeting one of the following criteria: 1) a physician diagnosis of COPD/emphysema, 2) a physician diagnosis of chronic bronchitis, or 3) a symptom-based definition of chronic bronchitis, plus either regular use of respiratory medication for their condition or chronic cough with phlegm most days. The survey was conducted between November 2012 and May 2013. Participation in the survey was entirely voluntary and confidential, and all subject data were anonymous. Prior to completion of the survey, all respondents were informed that they could terminate the interview at any time and their verbal consent to participate was recorded as part of the survey procedures. The survey protocol and consent procedure were reviewed by the Abt SRBI Institutional Review Board (IRB) (registered with the Office for Protection from Research Risks, Health and Human Services) and granted an IRB exemption as the criteria for exemption under 45 CFR 46.101(b)(2) of the United States Code of Federal Regulations were met.

Patients from 12 countries (Brazil, France, Germany, Italy, Japan, Mexico, the Netherlands [NL], Russia, South Korea [SK], Spain, the United Kingdom [UK], and the United States of America [USA]) provided responses to a structured questionnaire either by telephone or in face-to-face interviews. Patients were asked about their disease severity, symptoms, medications and COPD-related healthcare resource use (including hospitalisations, emergency department (ED) visits, and healthcare professional consultations). Several patient-reported instruments were completed including modified Medical Research Council (mMRC) Dyspnoea Scale [12], and the COPD Assessment Test (CAT) [13]. Patients were also questioned about their work loss due to COPD and completed the Work Productivity and Activity Impairment Questionnaire (WPAI) which measures impacts on work as a result of poor health [14]. Higher percentages indicate greater impairment and less productivity.

### Statistical analysis

A societal perspective was used which included COPD-related healthcare resource use and lost productivity. Costs were calculated using local country specific costs and converted to US dollars (2013) using the average historical exchange rates over the survey period (Table A in S1 Supporting Information).

#### Direct costs

Direct cost measures were estimated for moderate and severe COPD exacerbations, contact with healthcare professionals, COPD medications, home oxygen use and influenza vaccination during the past 12 months. For this analysis, exacerbations of COPD were defined as episodes of worsening breathing problems that required treatment with antibiotics and/or systemic corticosteroids. These were further categorized as moderate (community treated) which were costed as a general practice visit plus a course of prednisolone 40mg once daily for 5 days and co-amoxiclav 875mg/125mg twice a day for 7 days (in line with global COPD guidelines^1^) and severe (requiring an ED visit and/or hospitalisation) which were costed for the ED and/or inpatient stay as reported. Of note, the requirement of treatment in addition to self reported worsening of breathing problems to define exacerbations was implemented in this study to improve the specificity of the definition for an exacerbation. Due to this additional requirement, the reported percentage of patients with an exacerbation episode reported herein is slightly lower than previously reported for this survey.[11] Health care professional (HCP) visits were based upon the medical specialty seen most often by patients in the survey (either a GP/Specialist/Nurse) and the corresponding frequency of contact. For the 1% of HCP visits that were recorded as ‘don’t know’ or ‘refused’, we imputed the mostly common used HCP type from that country. In addition, if a HCP type was reported but the frequency of visiting that HCP was missing, the average frequency data from that country were imputed. For COPD medications, only patients that reported using a prescription medication in the past year (92%) were included in the cost analysis (fewer than 2% reported ‘don’t know’ or refused to respond to the medication question and there were no imputations for these data). All COPD prescription medicines reported were grouped by drug class; it was assumed that only one drug from the same class was taken at any time; drugs across different classes were assumed to be taken concurrently. All medications were assumed to be taken for a full year as per the licensed dose. Antibiotics and oral/systemic corticosteroids were included in the cost of moderate exacerbations and were therefore excluded from the treatment cost calculation to avoid double counting. The cost for each class was calculated using a weighted average (based on market share) of the five most commonly prescribed preparations within that class in each country (Table B in S1 Supporting Information). Patients who indicated that they had received an influenza vaccination were assumed to have received a single vaccination. For patients reporting home oxygen use, an average annual cost for their country was applied.

The annual per patient direct costs were calculated for each healthcare resource measure, by multiplying the frequency of use in the past year by unit costs obtained from local health economics experts in each participating country, global datasets or local prescribing data on medication sales. Each of these costs was combined to provide an estimate of the total annual mean direct cost of COPD per patient.

#### Indirect costs

Indirect costs were evaluated using the human capital approach, based on the equivalence between the value of lost productivity and the associated annual earning for obtaining this production [15]. Annual mean indirect costs were calculated by multiplying the mean percent of annual work loss by the annual average income (country specific) (Table C in S1 Supporting Information). Work loss in the week prior to the survey was estimated using WPAI and extrapolated to the prior 12 months, and was only considered in patients of working age (based on effective retirement age in each country (Table C in S1 Supporting Information). Patients who reported that they were retired and were aged below the effective retirement age for their country were assumed to have 100% work loss. As a scenario analysis, indirect costs were also calculated using the friction cost method. This method reduces the likelihood of overestimating indirect costs by assuming that a patient missing work due to a long period of illness is likely to be replaced by another employee, rather than the position remaining vacant until the patient is able to return to work [16]. A friction period of 90 days and an elasticity correction factor of 0.8 were used for all countries as these were the most frequently reported in the literature [17].

#### Total societal costs

Direct and indirect costs were combined to estimate the total annual societal cost of COPD per patient. A sensitivity analysis of total societal costs was also conducted, in which national currencies were converted into US dollars using purchasing power parities instead of exchange rates (Table A in S1 Supporting Information).

In order to identify subgroups of patients in which the burden of COPD was disproportionately high, total societal costs were compared across the following subgroups for each country:

- Breathlessness (mMRC)—mMRC 0–1, mMRC≥2 (moderate-to-severe dyspnoea)
- Health status (CAT)—CAT≤20, CAT>20 (high-to-very high impact)
- Self- perceived severity of COPD–mild, moderate, severe/very severe
- Adherence (Morisky MMAS-8)—Low adherence <6, medium adherence 6 to <8, high adherence ≥8
- Comorbidity—<2, ≥2 self reported co-morbidities from a list of 6 *a priori* comorbidities of clinical interest (asthma, hypertension, heart attack, heart failure, stroke, and cancer)

To minimize the effect of sex and age imbalances by country in the sampling and screening process, the reported percentages and costs were weighted by age and sex according to the latest census data available in each country.

## Results

### Patient demographics and baseline characteristics

A total of 4,343 respondents fulfilled the case definition of COPD and consented to complete the full survey. A total of 45% qualified with a physician diagnosis of COPD, chronic obstructive airway disease, or emphysema; 45% had a physician diagnosis of chronic bronchitis; and 10% had symptom-based chronic bronchitis. The mean age of the population ranged from 57.2 years (Russia) to 66.8 years (France), with Brazil reporting the highest proportion of patients of working age (75%) and France reporting the lowest (31%) (Table 1). The majority of patients in each country perceived their COPD as being mild or moderate. When COPD burden was assessed using validated measures, the frequency of moderate to severe dyspnoea (mMRC Dyspnoea Scale score ≥2) ranged from 25% in Japan to 61% in the UK, and the frequency of high-to-very high impact on health status (CAT score >20) ranged from 34% in Japan to 72% in Brazil. Japanese patients also had the lowest frequency of co-morbidity (16% reporting two or more co-morbid diseases) while a third or more of patients in the USA, Brazil, Germany and the Netherlands reported two or more comorbidities.

**Table 1:** Patient demographics and baseline characteristics: Continuing to Confront COPD International Patient Survey, 2012–13.

	USA n = 1,001	Mexico n = 328	Brazil n = 300	France n = 300	Germany n = 300	Italy n = 302	Spain n = 303	UK n = 305	NL n = 303	Russia n = 301	Japan n = 300	SK n = 300
**Female (%)**	52	57	55	52	56	43	43	56	51	61	46	40
**Age in years, mean (SD)**	60.9 (10.9)	62.1 (12.2)	57.9 (12.4)	66.8 (13.6)	59.7 (11.2)	63.1 (12.8)	65.1 (12.5)	62.4 (11.3)	61.7 (11.2)	57.2 (11.1)	59.6 (12.6)	61.8 (12.2)
**Working age (%)**	63	74	75	31	58	43	40	53	53	54	74	72
**Self-reported COPD severity (%)**												
**Mild**	30	16	21	25	31	26	27	24	28	26	59	14
**Moderate**	41	56	55	57	41	56	56	41	54	67	28	53
**Severe/very severe**	29	27	24	19	28	18	17	35	19	7	13	33
**mMRC grade, %**												
**0–1**	42	42	40	50	58	40	49	37	65	60	69	51
**≥2**	50	50	56	43	36	47	44	61	30	38	25	46
**Missing**	8	8	4	8	6	13	7	3	5	2	6	3
**CAT score,%**												
**≤20**	33	34	27	56	51	49	58	28	60	42	66	31
**>20**	60	66	72	42	46	49	38	66	34	56	34	69
**Missing**	7	0	1	2	3	2	4	7	6	2	1	0
**Comorbidities, %**												
**0–1**	47	76	60	70	66	71	69	74	67	74	84	73
**≥2**	53	17	39	28	34	28	30	26	32	24	16	26
**Missing**	0	7	1	2	0	1	1	0	1	2	0	1

Abbreviations: CAT, COPD Assessment Test; COPD, chronic obstructive pulmonary disease; SD, standard deviation; mMRC, modified Medical Research Council Scale; NL, Netherlands; South Korea, SK.

### Healthcare resource use

With respect to COPD-related healthcare resource use, patients across all countries except Japan and Brazil reported more frequent contact with general practitioners (GPs) than with specialists (Table 2). The mean number of contacts with GPs in the prior 12 months ranged from 2.6 (SD = 5.2) in Spain to 8.3 (SD = 11.9) in the UK, while specialists were seen on average from 1.6 visits (SD = 4.4) in Russia to 5.1 visits (SD = 8.8) in Japan.

**Table 2:** Frequency of contact with healthcare professionals and frequency of exacerbations: Continuing to Confront COPD International Patient Survey, 2012–13.

	USA n = 1,001	Mexico n = 328	Brazil n = 300	France n = 300	Germany n = 300	Italy n = 302	Spain n = 303	UK n = 305	NL n = 303	Russia n = 301	Japan n = 300		SK n = 300
**General practitioner (GP) contact**													
**Usual care from a GP (%)**	66	74	49	78	62	70	58	81	71	80	50		54
**Mean visits per patient (SD)**	4.4 (8.0)	7.4 (9.4)	4.2 (8.3)	6.5 (10.2)	4.2 (8.5)	4.6 (7.5)	2.6 (5.2)	8.3 (11.9)	4.1 (9.0)	4.5 (7.7)	5.1 (7.7)		5.7 (10.7)
**Specialist contact**													
**Usual care from a specialist (%)**	33	26	51	22	38	30	42	15	26	20	50		46
**Mean visits per patient (SD)**	2.4 (6.3)	2.6 (6.9)	4.0 (8.3)	2.3 (6.2)	3.3 (7.7)	2.0 (5.5)	2.8 (7.6)	2.3 (7.8)	1.7 (5.6)	1.6 (4.4)	5.1 (8.8)		4.9 (8.1)
**Moderate Exacerbations**^1^													
**At least one in past year (%)**	43	75	31	40	52	55	47	63	38	52	39	52	
**Mean number per patient (SD)**	1.4 (2.5)	2.7 (3.2)	1.0 (2.0)	1.0 (1.8)	1.6 (2.6)	1.0 (1.4)	1.0 (1.5)	1.9 (2.2)	1.1 (2.2)	1.4 (2.3)	2.4 (4.1)	4.1 (4.9)	
**Emergency department visit for exacerbation**													
**At least one in past year (%)**	19	21	50	9	8	6	31	18	9	19	7	3	
**Mean number per patient (SD)**	0.4 (1.2)	0.5 (1.5)	1.3 (1.9)	0.2 (0.9)	0.2 (0.9)	0.1 (0.5)	0.6 (0.9)	0.4 (0.9)	0.1 (0.5)	0.5 (1.5)	0.1 (0.4)	0.1 (0.5)	
**Hospitalization for exacerbation**													
**At least one (%)**	12	14	20	8	9	6	16	15	7	14	3	5	
**Mean number per patient (SD)**	0.2 (0.7)	0.3 (0.8)	0.4 (1.1)	0.2 (1.1)	0.1 (0.5)	0.1 (0.3)	0.2 (0.5)	0.3 (0.9)	0.1 (0.4)	0.2 (0.7)	0.1 (0.4)	0.1 (0.6)	

^1^Community-treated exacerbation consisting of a general practice visit plus a course of prednisolone 40mg once daily for 5 days and co-amoxiclav 875mg/125mg twice a day for 7 days.

Abbreviations: GP: general practitioner; ED: emergency department; SD: standard deviation; NL, Netherlands; SK, South Korea

The proportion of patients reporting at least one moderate exacerbation was highest in Mexico (75%), the UK (63%) and Italy (55%), and lowest in Brazil (31%) (Table 2). The mean number of moderate exacerbations ranged from 1.0 (Brazil [SD = 2.0]; France [SD = 1.8]; Italy [SD = 1.4]; and Spain [SD = 1.5]) to 4.1 (SD = 4.9) (South Korea) with the majority of individual countries reporting an approximate mean number in the range of 1–2. Mean numbers of severe exacerbations resulting in an ED visit or hospitalisation were noticeably higher in Brazil (mean [SD]: 1.3 [1.9] and 0.4 [1.1] respectively) compared with other countries.

The majority of patients (range 81% in Japan to 97% in Mexico) reported taking a prescription medication for COPD during the past year (Table 3). With respect to prescribing patterns of individual medications, there were wide variations across countries. Of note, patients in Russia and South Korea reported the most frequent use of over the counter and herbal remedies (Russia [67%], South Korea [35%]).

**Table 3:** Proportion of patients (%) reporting treatment with COPD medications, home oxygen use, and influenza vaccination: Continuing to Confront COPD International Patient Survey, 2012–13.

	USA n = 1,001	Mexico n = 328	Brazil n = 300	France n = 300	Germany n = 300	Italy n = 302	Spain n = 303	UK n = 305	NL n = 303	Russia n = 301	Japan n = 300	SK n = 300
**Prescription medication use (%)**												
**Any prescription medication for COPD in the past year**	92	97	93	80	95	95	94	95	93	90	81	91
**SABA and/or SAMA**	63	56	51	54	46	35	46	60	49	25	20	7
**LABA**	2	6	7	17	28	5	9	10	13	12	8	2
**LAMA**	27	8	12	10	22	12	26	21	28	1	21	7
**ICS**	12	12	10	20	17	16	11	17	28	20	20	7
**ICS/LABA**	47	0	18	7	24	23	36	27	50	21	38	20
**Xanthines**	2	7	4	3	9	1	3	2	1	16	20	5
**Roflumilast**	1	0	3	0	3	2	1	0	0	8	0	3
**Other COPD-related prescription medications**^1^	28	77	57	32	34	37	43	32	20	80	46	62
**Over the counter/herbal remedies**	14	24	16	5	32	5	8	7	18	67	8	35
**Influenza vaccination in the past year**	67	57	51	41	45	47	56	66	70	9	46	80
**Home oxygen therapy use in the past year**	27	17	16	20	8	12	13	14	6	7	5	4

^1^Includes: oral and systemic corticosteroids, antibiotics, cough medicines, leukotriene receptor antagonists.

Abbreviations: SABA, short acting beta-2 agonists; SAMA, short acting anti-muscarincs; LAMA, long acting anti-muscarincs LABA, long acting beta-2 agonists; ICS, in haled corticosteroid; NL, Netherlands; SK, South Korea

### Direct costs

**Fig 1:**
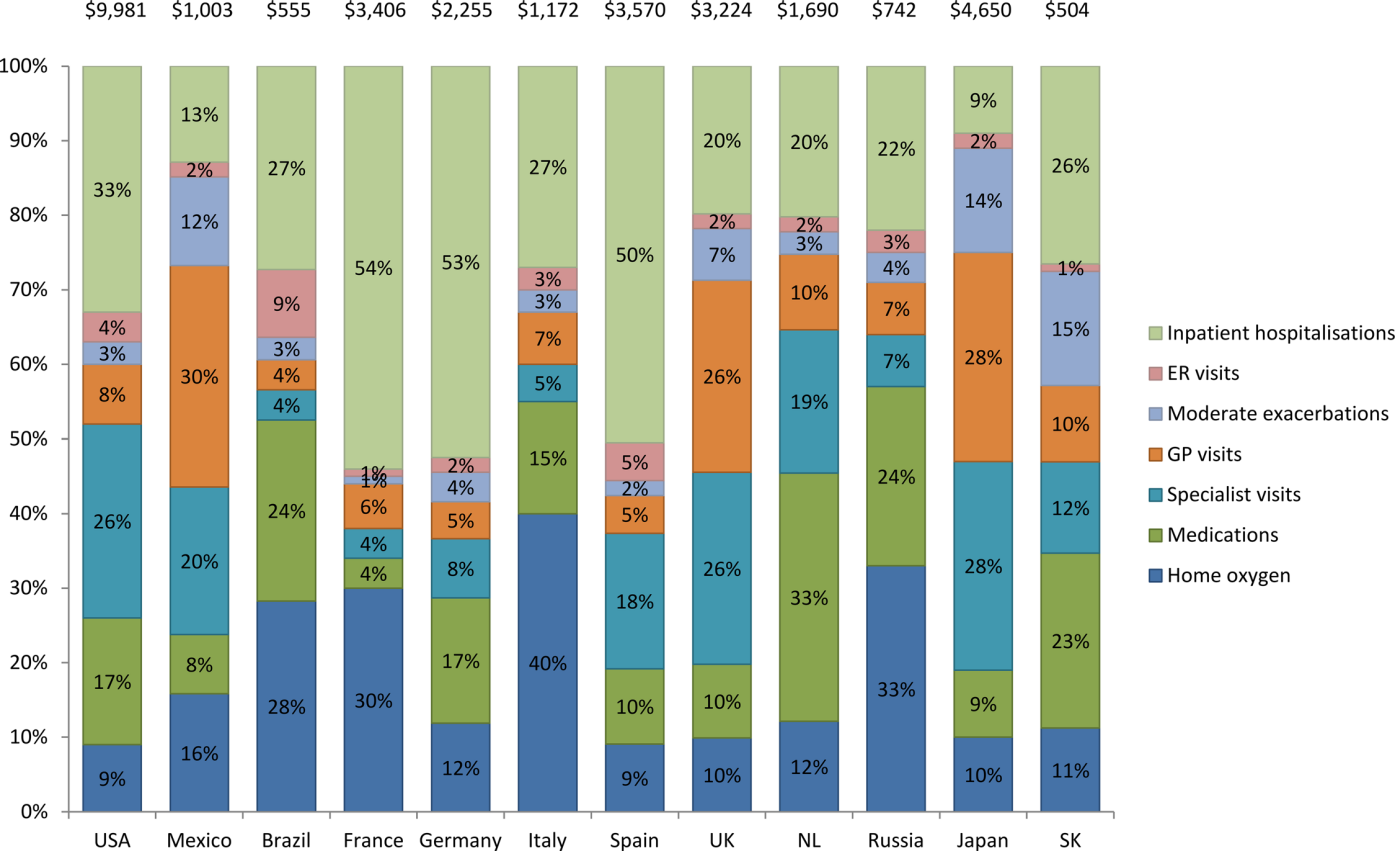
Annual direct costs per patient of COPD and percentage breakdown by cost type: Continuing to Confront COPD International Patient Survey, 2012–13. Additional costs not shown: Nursing visits: NL (1%); Influenza vaccination: Brazil (2%); SK (2%); NL (1%); Abbreviations: USA, United States of America, UK, United Kingdom, NL, Netherlands; SK, South Korea

The annual direct cost of COPD per patient ranged from $504 in South Korea to $9,981 in the USA (Fig 1). The breakdown of direct costs varied across countries. In five countries inpatient hospitalisations was the largest contributor to direct costs: France [54%], Germany [53%], Spain [50%], the USA [33%], and South Korea [26%]) (Fig 1). General Practitioner visits contributed substantially to the direct costs in Mexico (30%), Japan (28%) and the UK (26%) whilst specialist visits contributed at least one quarter of the direct costs in Japan (28%), the UK (26%), and the USA (26%). Home oxygen therapy accounted for the largest individual direct costs in Brazil (28%), Russia (33%), France (30%), and Italy (40%). Prescription medication costs for COPD accounted for between 4 and 33% of costs across countries, being the lowest in France (4%), Mexico (8%) and Japan (9%), and the highest in Brazil (24%), Russia (24%) and the Netherlands (33%).

### Lost work productivity and indirect costs

**Fig 2:**
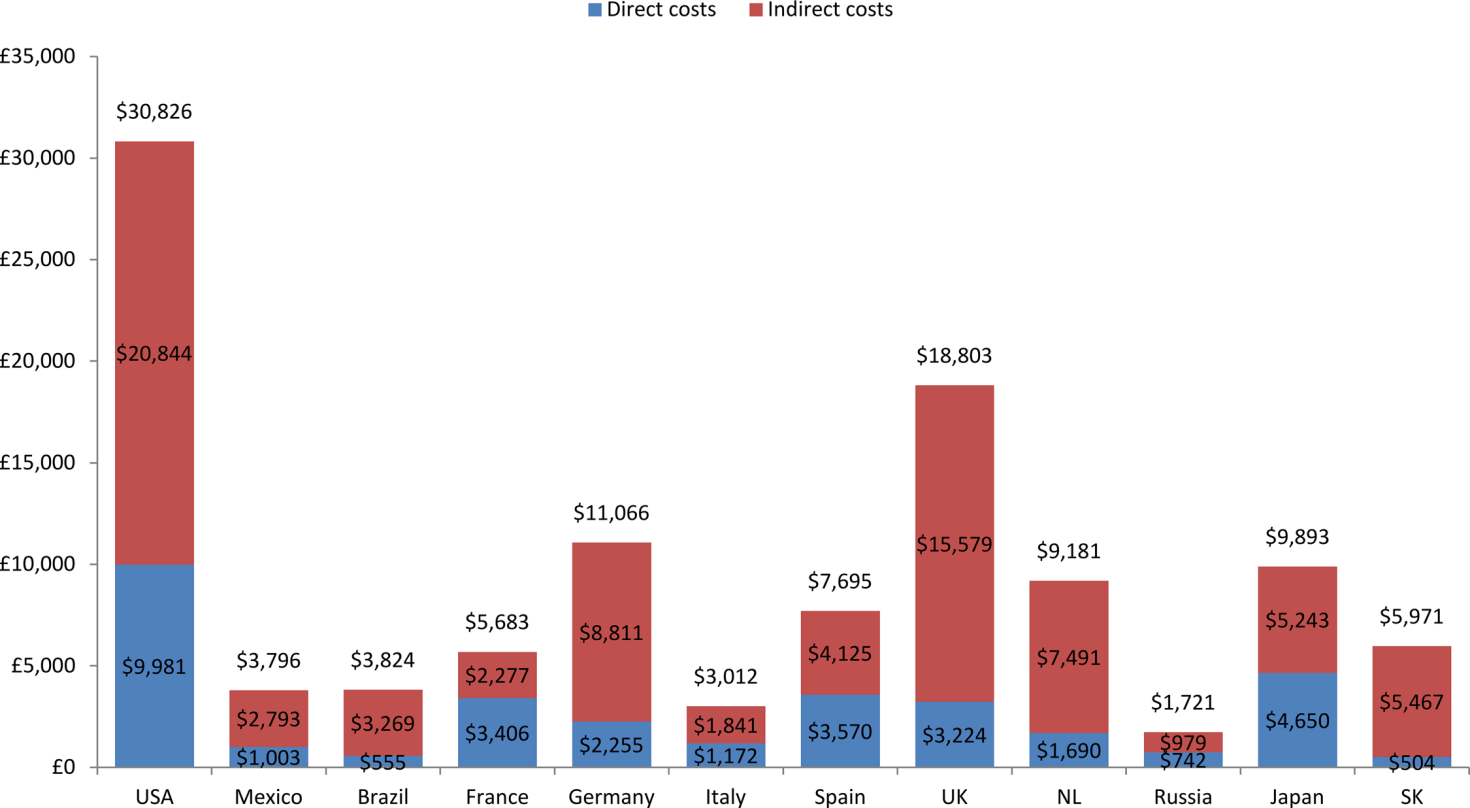
Annual societal costs per patient of COPD in each country (using exchange rates): Continuing to Confront COPD International Patient Survey, 2012–13. Abbreviations: USA, United States of America, UK, United Kingdom, NL, Netherlands; SK, South Korea

The proportion of working age patients completely prevented from working due to their COPD ranged from 6% to 52% across countries with the highest proportions reported in the UK and the USA (52%) and the lowest (≤10%) reported in Italy, Russia and Japan (S1 Fig). Patients reporting a limited ability to work ranged from 11% (Brazil and the Netherlands) to 28% (Germany), with the majority of countries falling within the range 11% to 19% (S1 Fig). The annual indirect cost of COPD ranged from $979 (Russia) to $20,844 (the USA) (Fig 2). Indirect costs calculated using the friction cost method were around a quarter of those calculated using the human capital approach (Table D in S1 Supporting information).

### Total societal costs

**Fig 3:**
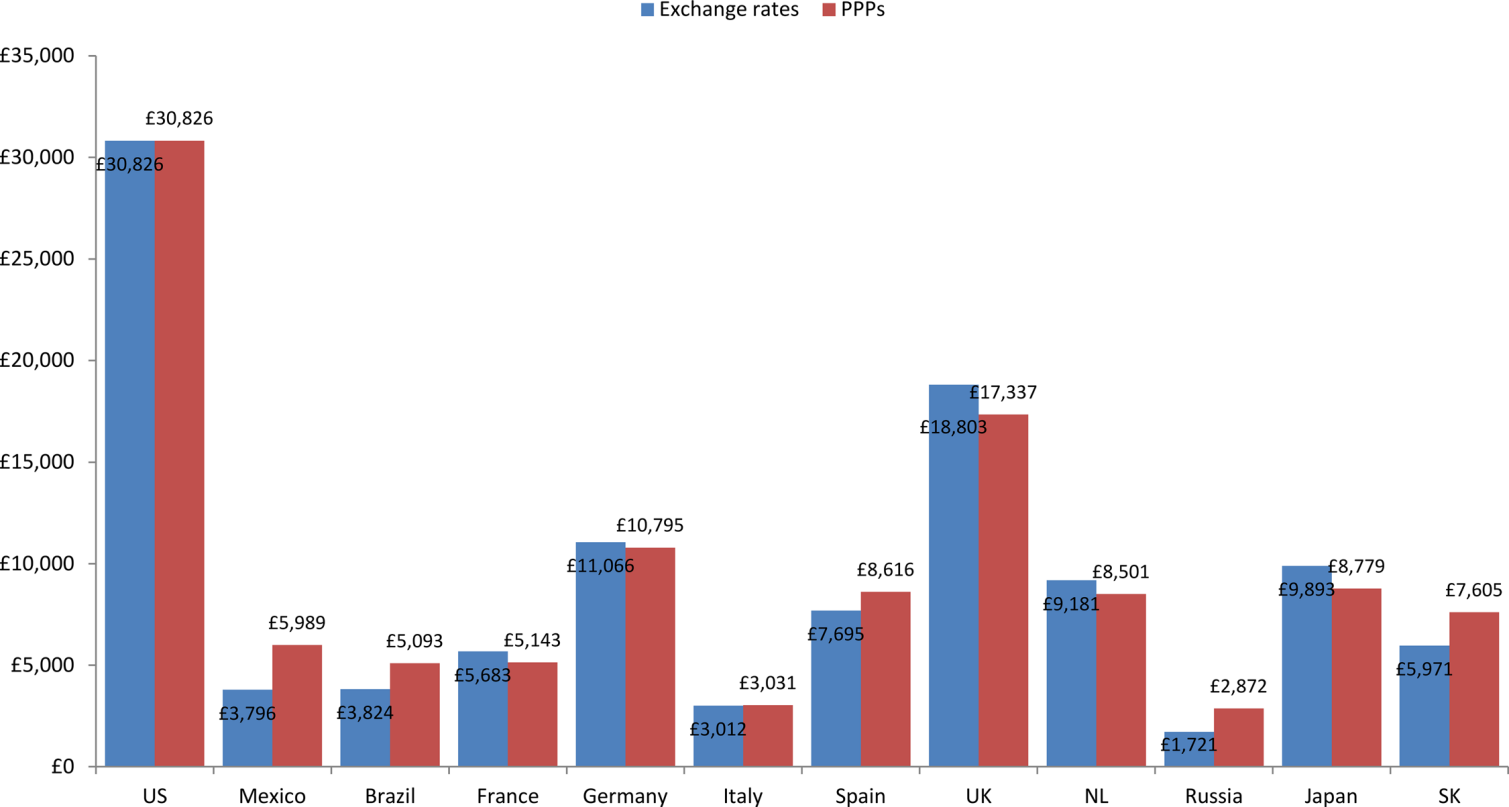
Annual societal per patient cost using exchange rates (Nov 2012 to May 2013) and Purchasing Power Parities (2013) (US$): Continuing to Confront COPD International Patient Survey, 2012–13. Abbreviations: USA, United States of America, UK, United Kingdom, NL, Netherlands; SK, South Korea

The calculated annual societal per patient cost of COPD varied widely across countries, ranging from $1,721 in Russia to $30,826 in the USA (Fig 2). The indirect cost of COPD exceeded the direct cost in all countries except for France, where indirect costs per patient were $2,277 and direct costs per patient were $3,406. Indirect costs in Brazil, Germany, the UK, the Netherlands and South Korea comprised over 80% of the total societal costs. A sensitivity analysis of total societal costs using purchasing power parities instead of exchange rates demonstrated higher per patient costs of COPD in Mexico, Brazil, Russia, Spain, and South Korea, and similar or slightly lower costs in the remaining countries outside of the USA (Fig 3). However, the order of magnitude of overall societal costs across countries remained largely the same.

**Fig 4:**
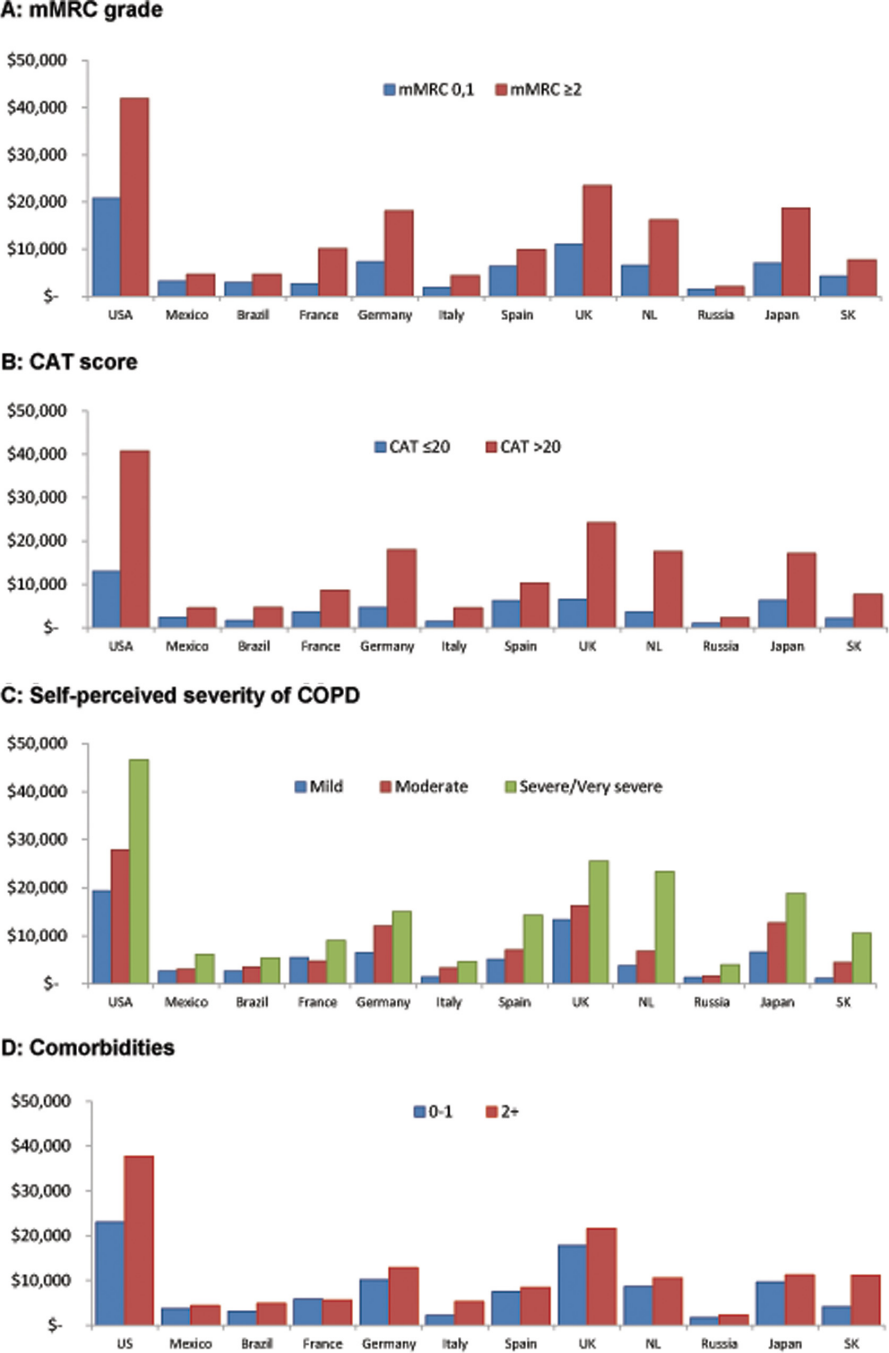
Annual societal costs per patient using exchange rates of COPD stratified by disease and patient characteristics: Continuing to Confront COPD International Patient Survey, 2012–13. (A) By mMRC grade. (B) By CAT score. (C) By self-perceived severity of COPD. (D) By Comorbidities. Abbreviations: CAT, COPD Assessment Test; mMRC, modified Medical Research Council Scale; USA, United States of America, UK, United Kingdom, NL, Netherlands; SK, South Korea

The total societal costs per patient were greater in patients who reported increased levels of breathlessness (mMRC ≥2), greater impact on health status (CAT score >20), and perceived themselves to have more severe COPD, than in those reporting less symptoms and less severe disease (Fig 4). These findings were consistent across all countries. Patients who reported having two or more comorbidities were slightly more costly than patients with none or one comorbidity, across most countries (an exception was France where costs were similar) (Fig 4).

## Discussion

This analysis of data from the Continuing to Confront COPD International Patient Survey evaluated the economic impact of COPD in a global population of patients across 12 countries. This dataset provides one of the largest global cost of illness evaluations to date and, importantly, allows for a comparison with the original Confronting COPD Survey, enabling a study of the changing economic impact of COPD over the last decade.

The current survey showed that the economic burden of COPD is considerable in all countries studied. The annual societal costs per patient covered a wide span ranging from $1,721 (Russia) to $30,826 (USA); however costs for the majority of countries fell within the approximate range of $4,000-$11,000. The largest direct cost associated with COPD in 5 of the 12 countries was attributed to hospitalisations. Other healthcare resources that made the largest contribution to COPD direct costs were: home oxygen therapy (3 countries), GP or specialist visits (equal contribution in 2 countries), GP visits (1 country), and medication costs (1 country). Indirect costs including productivity loss were several times that of direct costs in many countries including the USA, Germany, the UK, the Netherlands and South Korea. This highlights the “hidden” burden of COPD often ignored by decision makers whilst making policy decisions.

The differing local unit costs of healthcare services in each country had limited impact on the variability across different countries as shown by consistency in costs estimated using exchange rates and purchasing power parities. Variability in the COPD patients surveyed across multiple countries also had limited impact on societal costs with no consistent pattern observed. For example, the USA had high proportion of severe/very severe COPD patients and patients with two or more comorbidities consistent with high direct COPD costs. In contrast, Japan had high proportion of patients with mild disease and yet had the second highest direct costs. Direct costs were likely to be driven by healthcare systems and patients’ access to healthcare. Developed countries where access to COPD care was often free at the point of delivery tended to have higher direct costs. Patients in these countries also tended to have high indirect costs, mainly driven by higher national per capita incomes. Other factors influencing variations in cost data could be those related to cultural and healthcare practices differences. Patients in Japan reported the lowest levels of symptoms but had one of the highest proportions treated under specialist care.

A consistent finding across countries was the association between increased reported burden of COPD (breathlessness, symptoms and comorbidities) and greater total societal costs per patient. These findings are consistent with those reported in previous international surveys of COPD patients in developed and developing countries [6–8], and in a recent systematic review highlighting the substantial humanistic and economic burden of symptomatic COPD [18]. This further emphasizes the opportunity to impact the economic burden of COPD through better symptom control and the effective management of comorbidities. There were no consistent differences between costs in current smokers, former smokers and those who had never smoked (data not shown). This differed from the original Confronting COPD survey which reported that former smokers were more costly to healthcare systems than current smokers, and which the authors suggested could have been related to smokers quitting upon developing disease symptoms or immediately after diagnosis of smoking-related diseases [6].

Comparing the cost of COPD across individual studies, a USA medical expenditure survey in 2010 reported an average per person medical expenditure on COPD to be $9,800 (compared with $30,826 in the current survey) which included direct medical expenses and costs due to work loss (absenteeism), but not costs due to lost work productivity (presenteeism) which were included in the Continuing to Confront COPD Survey [19]. A prospective one-year follow-up study of primary care patients in Spain from 1996 to 1997 reported direct annual costs of $1,876, compared with $3,570 in the current survey, and concurring with the present findings, the biggest single contributor to costs was hospitalisations [20]. Nishimura *et al* used an economic model to assess the economic impact of COPD in Japan using data published between 1990 and 2002, and estimated the average annual total cost per patient for moderate/severe COPD to be US$3,694, compared with $9,893 in the Continuing to Confront COPD Survey, although 80% of costs were attributed to direct costs and 20% to indirect costs while the cost contribution was relatively equal in the current analysis [21]. Interestingly, the Nishimura study reported that outpatient visits accounted for 50% of direct costs, very similar to the findings in the current survey. A 2009 study conducted in the South Korean National Health Insurance database estimated direct costs per patient of $2,803, greater than the $504 in the current survey, although the Kim *et al* study population were receiving at least two COPD medications and also included intensive care and tertiary medical care costs [22]. A cross-sectional survey of physician-diagnosed COPD patients in Brazil, China, Germany, Turkey, the UK and the USA, reported lower indirect costs due to working hours lost than that shown in the Continuing to Confront COPD survey, but the magnitude of losses showed a similar pattern by country with the greatest impacts reported in the UK and the USA [6]. However these cross-study comparisons are limited due to differences in methodologies, populations studied and the type of economic evaluation used.

A main strength of the Continuing to Confront COPD Survey is that it allows for the assessment of change in the economic burden of COPD over a decade since the original Confronting COPD Survey was undertaken. In countries that participated in both surveys (France, Italy, the Netherlands, Spain, the UK and the USA), the annual societal costs per patient has increased above inflation in all countries [6]. The original Confronting COPD International Survey reported that the main driver of direct costs was inpatient hospitalisations in 5 out of 7 countries surveyed, and that the country incurring the biggest direct costs was the USA, the latter being consistent with the updated findings. The original survey reported high direct costs in Spain and low direct costs in the Netherlands which was also observed in the current analysis. In contrast, while France had the lowest direct costs in the original survey, it is among the highest for Europe in the current survey. This may be due to a higher contribution of inpatient hospitalisations to the French direct costs in the current survey compared to a decade ago. There was also consistency between surveys in the specific countries reporting high levels of indirect costs (USA, UK and the Netherlands). However, the use of the WPAI questionnaire in the current survey may have contributed to the observation that the indirect costs were higher than direct costs in nearly all countries, which is in contrast to the original Confronting COPD survey. Whilst the friction cost method is not widely used outside of the Netherlands [17], applying it as a scenario analysis for all countries resulted in a ratio of indirect to direct costs that more closely resembled what was seen in the original survey (where the friction cost method was only applied in the Netherlands).

In addition to historical comparison, it can also be of interest to understand COPD costs in the context of other common chronic diseases. A review by Muka *et al* ranked the costs associated with COPD to be lower than those for cancer and cardiovascular disease and higher than those associated with diabetes [23]. For example, direct costs for type 2 diabetes mellitus based on a systematic review of the literature from 2001 to 2014 ranged from $242 to $11,917 and indirect costs ranged from $45 to $16,914 [24]. We found indirect costs were higher than direct costs across all countries except for France, which has also been suggested in other disease areas. A literature review investigating the economic impact of a range of non-communicable diseases (including coronary heart disease, stroke, type 2 diabetes mellitus, cancer, COPD and chronic kidney disease) on work productivity, reported that people with diabetes, COPD and survivors of breast and lung cancer were at the highest risk of impact on work productivity [25].

The case definition used in this study included a variety of conditions reflecting how COPD may be diagnosed in different countries, including both physician-diagnosed disease and chronic bronchitis based on symptoms. Diagnosis of COPD based on symptoms overcame some of the problems associated COPD under-diagnosis and with physicians using alternative diagnosis terms when speaking to their patients. We accept that it is possible that some patients with lung diseases other than COPD may have been included under this definition, but symptom based COPD represented only 10% of the final sample. Another limitation of the Continuing to Confront COPD International Patient Survey is that outcomes were self-reported and could not be clinically verified, although this was partly mitigated by the use of validated patient reported outcome instruments. Furthermore, resource use was derived indirectly from survey responses and assumptions inevitably needed to be made in order to bridge survey responses to resource counts. A fixed cost was assigned to each type of resource use based on information from local health economics experts in each participating country, global datasets or local prescribing data on medication sales. The accuracy of these cost estimates was fundamental to the resultant estimated costs in each country.

The survey attempted to collect a variety of costs attributable to COPD, however, it was not possible to capture every single resource use parameter (e.g. pulmonary rehabilitation, lung volume reduction surgery) or monetise every parameter recorded (e.g. cost of all medications or all healthcare professional visits). Costs of out-of- pocket expenses such as non-prescription medication were also excluded. This survey did not include indirect costs due to caregiver work loss, suggesting that indirect costs of COPD may be even higher than that reported. In addition, if retirement ages increase in the future, the burden due to early retirement could also be higher as was suggested by Fletcher *et al* in the COPD Uncovered International Survey in a working age COPD population [8]. Overall, this may lead to potential underestimation of the total economic burden attributable to COPD.

## Conclusion

This economic analysis of the Continuing to Confront COPD International Patient Survey showed that COPD continues to place a high economic burden on the healthcare system and society in all countries studied. Indirect costs were higher in most countries and, patients with breathlessness, symptoms and comorbidities experienced higher costs. Attempts to reduce the economic burden will require interventions aimed at delaying the progression of disease, preventing exacerbations, optimising medication usage and reducing the risk of comorbidities in patients with COPD. In addition, strategies to allow COPD patients to remain in work are important for addressing the substantial wider societal costs.

## Supporting Information

S1 Fig Percentage of patients of working age reporting lost productivity due to COPD: Continuing to Confront COPD International Patient Survey, 2012–13. Abbreviations: USA, United States of America, UK, United Kingdom, NL, Netherlands; SK, South Korea
(TIF)

S1 Supporting Information **Table A:** Historical average exchange rates (Nov 2012 to May 2013) and Purchasing Power Parities (2013) **Table B:** Country specific unit costs (US$, 2013) **Table C:** Effective retirement age and annual average income **Table D:** Indirect costs using the friction cost method
(DOCX)
