# Supplementary figures and images for: Continuing to Confront COPD International Patient Survey: Economic Impact of COPD in 12 Countries
Source: PLoS One. 2016 Apr 19;11(4):e0152618. doi: 10.1371/journal.pone.0152618 (PMC4836731; doi:10.1371/journal.pone.0152618)

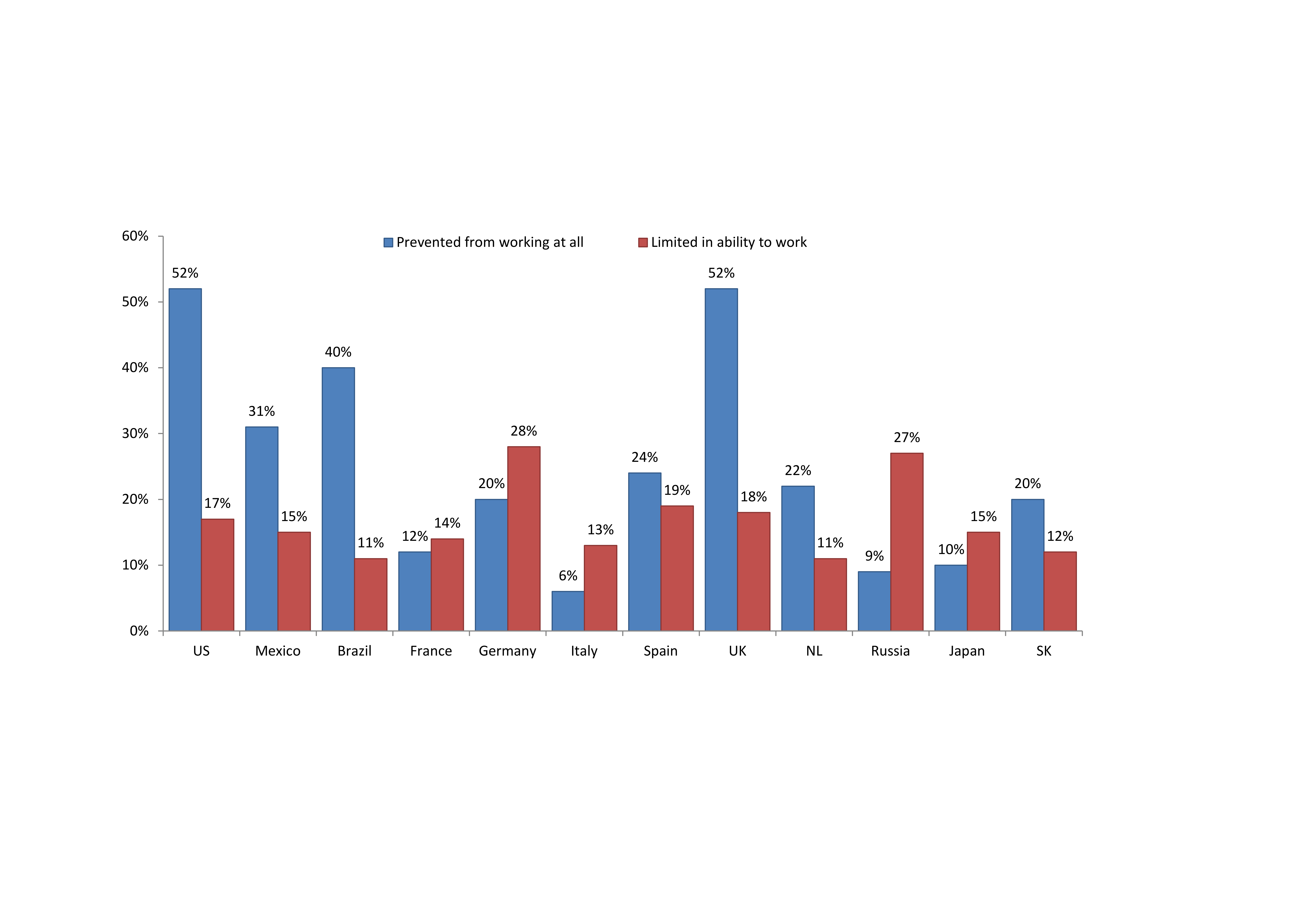

Supplement: S1 Fig — Abbreviations: USA, United States of America, UK, United Kingdom, NL, Netherlands; SK, South Korea (TIF) [file pone.0152618.s001.tif]
