# Supplementary material for: Continuing to Confront COPD International Patient Survey: Economic Impact of COPD in 12 Countries
Source: PLoS One. 2016 Apr 19;11(4):e0152618. doi: 10.1371/journal.pone.0152618 (PMC4836731; doi:10.1371/journal.pone.0152618)
Supplement: S1 Supporting Information — Table A: Historical average exchange rates (Nov 2012 to May 2013) and Purchasing Power Parities (2013) Table B: Country specific unit costs (US$, 2013) Table C: Effective retirement age and annual average income Table D: Indirect costs using the friction cost method (DOCX) [file pone.0152618.s002.docx]

**Continuing to Confront COPD International Patient Survey: Economic impact of COPD in 12 countries**

**Supporting information**

**Table A: Historical average exchange rates (Nov 2012 to May 2013) and Purchasing Power Parities (2013)**

|  | **USA** | **Mexico** | **Brazil** | **France** | **Germany** | **Italy** | **Spain** | **UK** | **NL** | **Russia** | **Japan** | **SK** |
| --- | --- | --- | --- | --- | --- | --- | --- | --- | --- | --- | --- | --- |
| Historical average exchange rates | 1 | 12.6221 | 2.0248 | 0.7648 | 0.7648 | 0.7648 | 0.7648 | 0.6408 | 0.7648 | 30.8604 | 91.4 | 1,092.85 |
| Purchasing Power Parities | 1 | 8 | 1.52* | 0.845 | 0.784 | 0.76 | 0.683 | 0.695 | 0.826 | 18.49* | 103 | 858 |
| NL, Netherlands; South Korea, SK | | | | | | | | | | | | |

**Table B: Country specific unit costs (US$, 2013)**

| **Healthcare Resource Measure** | **USA** | **Mexico** | **Brazil** | **France** | **Germany** | **Italy** | **Spain** | **UK** | **NL** | **Russia** | **Japan** | **SK** |
| --- | --- | --- | --- | --- | --- | --- | --- | --- | --- | --- | --- | --- |
| **A moderate (community treated) exacerbation** | 197.15 | 43.25 | 18.17 | 40.85 | 59.09 | 33.29 | 80.23 | 113.38 | 47.44 | 22.39 | 262.78 | 18.66 |
| **An emergency department visit (no admission)** | 946.09^1^ | 38.74^2^ | 39.52^3^ | 194.22^6^ | 248.95^c^ | 315.18^10^ | 346.41^f^ | 173.21^14^ | 215.74^16^ | 48.83^17^ | 751.07^g^ | 51.13^21^ |
| **A hospital episode** | 15,050.41^1^ | 450.32^2^ | 360.09^3,4^ | 10,806.75^7^ | 9,131.59^8,9^ | 4,057.19^11^ | 8,236.34^f^ | 2,136.38^14^ | 3,393.04^16^ | 750.09^18^ | 7,510.74^19^ | 1,072.70^21^ |
| **A GP visit** | 184.87^1^ | 40.01^2^ | 4.94^5^ | 31.46^7^ | 26.77^8^ | 17.70^12^ | 74.78 | 100.83^15^ | 40.53 | 10.98^17^ | 253.77^20^ | 8.89^21^ |
| **A nurse visit in primary care** | 184.87^a^ | 40.01^2^ | 3.11^5^ | 14.85^7^ | N/A | 17.70^a^ | 27.78^f^ | 35.21^15^ | 43.15^f^ | 10.98 ^a^ | N/A | 8.89 ^a^ |
| **A specialist visit in secondary care** | 1,102.68^1^ | 76.53^2^ | 4.94^5^ | 53.13^7^ | 52.50^8^ | 29.99^13^ | 221.15^f^ | 359.58^14^ | 193.51^f^ | 31.87^17^ | 253.77 ^a^ | 12.48^21^ |
| **Short-acting β_2_-agonist (SABA) and/or Short-acting muscarinic antagonist (SAMA) (6 inhalers annually)** | 203.65 | 18.91 | 36.24 | 26.12 | 15.45 | 20.60 | 14.14 | 17.98 | 27.25 | 14.79 | 91.57 | 17.88 |
| **Long-acting β_2_-agonist (LABA) (annual cost)** | 1,650.13 | 290.50 | 198.22 | 273.22 | 307.58 | 342.14 | 460.62 | 473.15 | 449.79 | 253.47 | 324.67 | 359.23 |
| **Long-acting muscarinic antagonist (LAMA) (annual cost)** | 2,439.41 | 347.84 | 362.46 | 296.95 | 448.66 | 228.60 | 440.11 | 555.36 | 593.90 | 763.45 | 544.54 | 408.78 |
| **Inhaled corticosteroid (ICS) (annual cost)** | 1,342.36 | 116.39 | 103.67 | 174.70 | 113.01 | 44.81 | 68.35 | 114.87 | 286.89 | 117.72 | 365.30 | 217.21 |
| **ICS/LABA (annual cost)** | 2,405.80 | 313.47 | 303.79 | 610.99 | 1,155.72 | 630.93 | 698.71 | 668.14 | 685.99 | 428.34 | 775.42 | 398.15 |
| **Xanthines (annual cost)** | 356.80 | 140.12 | 149.62 | 32.66 | 14.24 | 34.94 | 39.87 | 61.03 | 83.16 | 11.39 | 169.74 | 75.07 |
| **Roflumilast (annual cost)** | 2,043.54 | 746.56 | 565.04 | N/A | 290.12 | 700.04 | 575.49 | 653.14 | 638.14 | 453.78 | N/A | 502.39 |
| **Influenza vaccine (one dose)** | 18.93 | 3.73 | 16.46 | 8.03 | 19.74^d^ | 7.52 | 7.93 | 8.46 | 13.08^f^ | 4.05 | 33.54 ^f^ | 13.36 |
| **Mean cost of oxygen therapy for COPD patients** | 3,329.24 | 951.08^b^ | 980.39 ^b^ | 5,058.53 | 3,405.60^e^ | 3,723.49 | 2,615.32^f^ | 2,225.06 | 3,405.60^e^ | 3,405.60^e^ | 10,083.15^f^ | 1,317.66 |
| **Prednisolone 40mg od for 5 days** | 0.86 | 19.42 | 1.73 | 3.25 | 2.41 | 6.79 | 1.70 | 4.67 | 1.69 | 0.70 | 2.99 | 0.48 |
| **Co-amoxiclav 875mg/125mg bd for 7 days** | 11.42 | 22.65 | 11.50 | 6.15 | 29.91 | 8.80 | 3.75 | 7.88 | 5.21 | 10.70 | 6.03 | 9.30 |
| ^a^ Assumed to be the same cost as a GP visit. ^b^ Estimated at 10% of annual average income. ^c^ Average of France, Italy, Spain, the UK and the NL. ^d^ Price to wholesaler of Influsplit SSW. ^e^ Average of France, Italy, Spain, and the UK. ^f^ Provided by local health economics expert. ^g^ Estimated at 10% of a hospital episode. Superscript numbers correspond to references in Reference list; NL, Netherlands; South Korea, SK | | | | | | | | | | | | |

**Table C: Effective retirement age and annual average income**

|  | **USA** | **Mexico** | **Brazil** | **France** | **Germany** | **Italy** | **Spain** | **UK** | **NL** | **Russia** | **Japan** | **SK** |
| --- | --- | --- | --- | --- | --- | --- | --- | --- | --- | --- | --- | --- |
| **Effective (practical) retirement age – Male (years)** | 65.0 | 72.3 | 70.7 | 59.7 | 62.1 | 61.1 | 62.3 | 63.7 | 63.6 | 60.0 | 69.1 | 71.1 |
| **Effective (practical) retirement age – Female (years)** | 65.0 | 68.7 | 64.0 | 60.0 | 61.6 | 60.5 | 63.2 | 63.2 | 62.3 | 55.0 | 66.7 | 69.8 |
| **Annual average income (US$, 2013)** | 56,340.00 | 9,510.85 | 9,803.93 | 46,514.12 | 46,996.60 | 37,812.50 | 35,002.62 | 50,230.96 | 55,558.32 | 11,584.55 | 43,485.95 | 30,444.22 |
| *Official retirement age used as effective retirement age not available. NL, Netherlands; South Korea, SK | | | | | | | | | | | | |

**Table D: Indirect costs using the friction cost method**

|  | | **Country** | | | | | | | | | | | |
| --- | --- | --- | --- | --- | --- | --- | --- | --- | --- | --- | --- | --- | --- |
|  |  | **USA** | **Mexico** | **Brazil** | **France** | **Germany** | **Italy** | **Spain** | **UK** | **NL** | **Russia** | **Japan** | **SK** |
| **Friction cost 80% elasticity (Exchange rates) (US$, 2013)** | **Mean** | 4800 | 638 | 729 | 673 | 2550 | 624 | 1155 | 3609 | 1799 | 419 | 1508 | 1366 |
| **Friction cost 80% elasticity (PPP) (US$, 2013)** | **Mean** | 4800 | 1006 | 971 | 609 | 2488 | 628 | 1293 | 3327 | 1666 | 699 | 1338 | 1740 |
| NL, Netherlands; South Korea, SK | | | | | | | | | | | | | |

**References**

1. Blanchette CM, Dalal AA, Mapel D. Changes in COPD demographics and costs over 20 years. J Med Econ 2012; 15(6):1176-1182.

2. IMSS. Mexican Institute of Social Security Unitary Costs. 2013. Available from

<http://compras.imss.gob.mx/?P=imsscompro>

3. Ministério da Saúde. Datasus. 2014 Available from: http://www2.datasus.gov.br/DATASUS/index.php

4. Farias CC, Resqueti V, Dias FA, Borghi-Silva A, Arena R, Fregonezi GA. Costs and benefits of pulmonary rehabilitation in chronic obstructive pulmonary disease: a randomized controlled trial. Braz J Phys Ther 2014; 18(2):165-173.

5. Ministério da Saúde. Sistema de Informações Ambulatoriais do SUS (SIA/SUS). 2014.

6. Fédération Hospitalière de France. GACAH 2009. 2009 Available from: <URL:http://www.fhf.fr/Actualites/Finances-BDHF/comptabilite-analytique/GACAH-2009>.

7. Échantillon Généraliste de Bénéficiaires. 2014.

8. Menn P, Heinrich J, Huber RM, Jorres RA, John J, Karrasch S et al. Direct medical costs of COPD--an excess cost approach based on two population-based studies. Respir Med 2012; 106(4):540-548.

9. Freytag, Antje and Storz, Philipp and Hagenmeyer, Ernst-Günther and Höer, Ariane and Caeser, Manfred and Häussler, Bertram and Gothe, Holger, Direct Costs of Treatment of COPD in a German Sickness Fund. iHEA 2007 6th World Congress: Explorations in Health Economics Paper. Available at SSRN: <http://ssrn.com/abstract=993135>

10. Ministero della Salute. Proposta metodologica per la valutazione dei costi dell'emergenza. 2007 Available from: <URL:http://www.mattoni.salute.gov.it/mattoni/documenti/11_Valutazione_costi_dell_emergenza.pdf>.

11. SDO. Rapporto annuale sull'attività di ricovero ospedaliero dati 2010. 2010 Available from: <URL:http://www.salute.gov.it/imgs/C_17_pubblicazioni_1690_allegato.pdf>.

12. Garattini L, Castelnuovo E, Lanzeni D, Viscarra C, Gruppo di studio DYSCO VISITE. Durata e costo delle visite in medicina generale: il progetto DYSCO. Farmeconomia e percorsi terapeutici 2003;4 (2):109-114.

13. Agenzia Nazionale per i Servizi Sanitari Regionali. Tariffe delle prestazioni specialistiche ambulatoriali aggiornato al 31 dicembre 2009. 2009 Available from: <http://www.agenas.it/ricerca-sul-sito>

14. Department of Health. NHS reference costs: financial year 2011 to 2012. 2012 Available from: <URL:https://www.gov.uk/government/publications/nhs-reference-costs-financial-year-2011-to-2012>.

15. PSSRU. Unit Costs of Health and Social Care 2012. 2012. Available from: <http://www.pssru.ac.uk/project-pages/unit-costs/2012/>

16. Oostenbrink JB, Rutten-van Molken MP. Resource use and risk factors in high-cost exacerbations of COPD. Respir Med 2004; 98(9):883-891.

17. Program of state guarantees. 2014 Available from: <http://www.rg.ru/2012/10/26/zdorovie-dok.html>

18. Saint-Petersburg obligatory insurance data. 2014 Available from: <URL:http://www.spboms.ru/kiop/main?page_id=48>.

19. Motegi T, Yamada K, Kida K. [Cost analysis for inpatient therapy for patients with acute exacerbations of chronic obstructive pulmonary disease]. Nihon Kokyuki Gakkai Zasshi 2006; 44(11):787-794.

20. Nishimura S. Survey of cost for medical treatment (average of 1999-2001 data). Pharma Medica 23, 165-173. 2005.

21. HIRA. National Medical Service Fee List. 2014. <https://www.hira.or.kr/eng/>
